# Preclinical Models of Hepatocellular Carcinoma: Current Utility, Limitations, and Challenges

**DOI:** 10.3390/biomedicines12071624

**Published:** 2024-07-22

**Authors:** Antonio Cigliano, Weiting Liao, Giovanni A. Deiana, Davide Rizzo, Xin Chen, Diego F. Calvisi

**Affiliations:** 1 Department of Medicine, Surgery and Pharmacy, University of Sassari, 07100 Sassari, Italy; acigliano@uniss.it (A.C.); gdeiana@uniss.it (G.A.D.); drizzo@uniss.it (D.R.); 2 Department of Bioengineering and Therapeutic Sciences and Liver Center, University of California, San Francisco, CA 94143, USA; liaoweit@hawaii.edu (W.L.); xinchen3@hawaii.edu (X.C.); 3 Cancer Biology Program, University of Hawaii Cancer Center, Honolulu, HI 96813, USA

**Keywords:** liver cancer, genetically engineered mice, hydrodynamic gene delivery, organoids, signaling pathways, precision medicine

## Abstract

Hepatocellular carcinoma (HCC), the predominant primary liver tumor, remains one of the most lethal cancers worldwide, despite the advances in therapy in recent years. In addition to the traditional chemically and dietary-induced HCC models, a broad spectrum of novel preclinical tools have been generated following the advent of transgenic, transposon, organoid, and in silico technologies to overcome this gloomy scenario. These models have become rapidly robust preclinical instruments to unravel the molecular pathogenesis of liver cancer and establish new therapeutic approaches against this deadly disease. The present review article aims to summarize and discuss the commonly used preclinical models for HCC, evaluating their strengths and weaknesses.

## 1. Introduction

**Figure 1:**
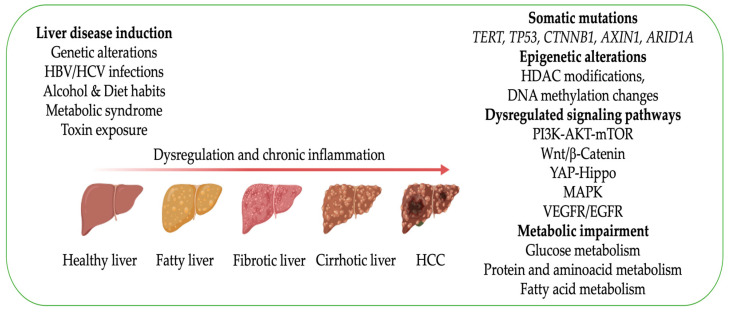
The multistep process leading to HCC development. This image was modified using the BioRender online tool (www.biorender.com, accessed on 10 June 2024). Abbreviations: HBV, hepatitis B virus; HCV, hepatitis C virus; TERT, telomerase reverse transcriptase; TP53, tumor protein 53; CTNNB1, catenin beta 1; ARID1A, AT-rich interaction domain 1A; HDAC, histone deacetylase; PI3K, phosphoinositide 3-kinase; MAPK, mitogen-activated protein kinase; VEGFR, vascular endothelial growth factor receptor; EGFR, epidermal growth factor receptor.

Hepatocellular carcinoma (HCC) is the most common primary liver tumor, accounting for ~90% of malignancies originating in this organ [1]. In addition, epidemiologic evidence indicates that HCC is the third leading cause of cancer-related mortality worldwide [2]. The major risk factors for HCC include chronic alcohol consumption, diabetes- or obesity-related NASH, and chronic infections by HBV or HCV [1]. While surgical resection, liver transplantation, and locoregional treatments are effective therapies against early-stage HCC, the management of patients with advanced HCC has been recently improved by combining immune checkpoint and tyrosine kinase inhibitors [3,4,5]. However, only a few (15–20%) patients benefit from this therapeutic strategy [4,5,6]. In the last decade, several molecular classifications of HCC have been proposed, revealing the existence of various and distinct HCC subsets [7,8]. This molecular heterogeneity is presumably responsible for the different responses of HCC patients to the available targeted therapies, underlining the need for valuable tools to elucidate each tumor subgroup’s peculiarities in driver signaling cascades and therapy sensitiveness. Therefore, it is imperative to establish sufficient preclinical models to investigate the molecular pathogenesis of HCC and evaluate the efficacy of novel therapies against this aggressive tumor (Figure 1).

The commonly used preclinical models for HCC include those induced by chemotoxic agents, special diets, genetic modifications, and tumor cell transplantation [9,10]. The present article provides an overview of the current preclinical models of HCC, summarizes their utility and limitations, discusses the challenges and opportunities for developing more effective preclinical models of HCC, and provides an outlook for the future of these models. A summary characterization of in vitro and in vivo preclinical models described in the present manuscript is presented in Table 1.

## 2. In Vitro HCC Models

### 2.1. Two-Dimendional (2D) Cell Lines 

To date, about 30 publicly available HCC cell lines exist (Table 2), and the genetic characteristics of these cell lines are largely undetermined [11]. Common human HCC cell lines include Hep3B, Huh7, SMMC-7721, SNU449, SNU182, MHCC97H, PLC/PRF/5, etc. These cells have been extensively used as a model system for studying liver cancer and evaluating potential therapies [11]. Major applications are related to molecular target discovery, drug screening, and in vivo xenograft development. Notably, the various cell lines respond differently to the same treatments, thus mimicking the heterogeneity of HCC patients’ response to the available drugs [12]. Moreover, a recent study used gene expression profile analysis of several HCC cell lines to provide a relevant criterion when selecting suitable cell lines for a specific study or objective [13]. 

**Figure 2:**
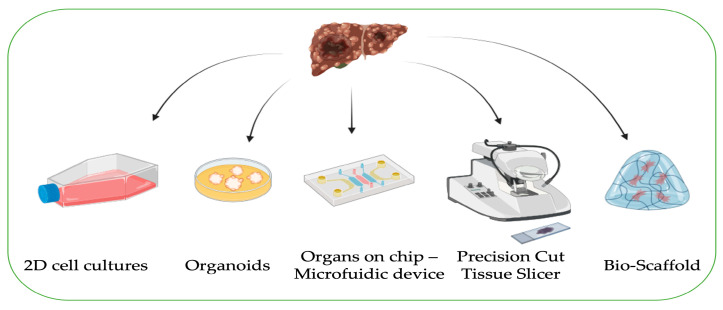
Overview of the in vitro models to study HCC. This image was created using the BioRender online tool (www.biorender.com, accessed on 10 June 2024).

In liver tumor research, 2D cell lines play an important role. Yet, the major limitation and disadvantage is not being able to fully represent the three-dimensional (3D) structure and complexity of the tumor, including the tumor microenvironment. In addition, 2D cells are derived from fully malignant tumors, which are generally poorly differentiated. Thus, the study of preneoplastic phases of hepatocarcinogenesis is not feasible in this system. For these reasons, other models, such as organoids, organ-on-chip, tissue slices, and bio-scaffolds, can be associated with traditional 2D studies to better understand the molecular features characterizing the tumor and its complexity (Figure 2).

### 2.2. Organoids

Models based on primary cell lines derived from HCC patients are rare. Indeed, the establishment of human cell lines from fresh patients’ tumors is technically tricky, partly due to the low viability of the freshly isolated HCC cells. Additionally, individual differences in tumors from patients could largely affect experimental results. 

The advent of 3D culture systems has made it possible to partially recapitulate the complexity and function of mammalian tissue in vitro by forming structures that resemble an adult organ in culture; these systems have been termed “organoids” [14]. Initially, organoids were defined as culture systems of primary tissue maintaining the three-dimensional structure and preserving architecture, physiological aspects, and organ properties, such as self-organization, multilineage differentiation, and histological features. Therefore, intestinal, liver, pancreas, stomach, and kidney organoids have been developed from pluripotent stem cells to elucidate tissue development, organogenesis, and stem cell behavior [14,15].

Moreover, organoids became promising disease models, and cancer organoids revealed themselves as a critical tool for better understanding carcinogenesis, metastasis formation, drug screening, and resistance to therapy to discover more in-line, personalized anticancer treatments [41]. Organoids, with their unique features such as long-term culturing, cryopreservation, and genetic manipulation, offer several strengths similar to 2D cultures. However, they lack organ crosstalk, stroma, and infiltrating immune cells, which are present in in vivo models. [42]. To bridge this gap, co-culture systems have been developed and play a crucial role in studying the interactions between tumor cells and components of the TME [43,44]. 

In 2017, Laura Broutier et al. successfully cultivated eight primary liver cancer (PLC)-like organs from liver cancer tissues derived from PLC patients. This groundbreaking research not only proved the feasibility of the long-term cultivation of PLC-like organs but also opened up exciting possibilities for recapitulating in a culture dish the tissue structure, expression profile, genomic landscape, and in vivo tumorigenicity of parental tumors [45]. As for stem cells, Lgr5+ liver stem cells and human iPSCs have been used. Single mouse Lgr5+ liver stem cells can be expanded as epithelial organoids in vitro and differentiated into functional hepatocytes in vitro and in vivo [46]. Clonal long-term expansion of primary adult liver stem cells opens experimental avenues for disease modeling, toxicology studies, regenerative medicine, and gene therapy [47].

Human liver tumoroids could be sourced from surgical specimens, suitable for early-stage HCC that could be cured by surgical resection [45]. However, surgical resection is not a treatment option for most patients with HCC when diagnosed at a late stage, and liver biopsy is not required for diagnosis in advanced cases with classic imaging findings [48]. In 2018, Sandro Nuciforo et al. successfully cultured liver cancer-like organs from needle samples of advanced liver cancer patients and identified them after 32 weeks of continuous cultivation. HCC tumoroids were established from patient-derived needle biopsies with around a 30% success rate, maintaining the genomic characteristics and genetic heterogeneity of the primary tumor and retaining the morphology and expression patterns of liver cancer [48].

In addition to human-derived HCC tissue, Cao et al. used DEN-induced liver tumors to culture mouse liver tumor organoids. Small organoids could be visualized after days 2–7; passage was required at around 7–14 days. The efficiency for successfully establishing an organoid model is around 70% [49]. The main reason for establishment failure is that some organoids cease to proliferate at an early stage due to extensive necrosis. The successful organoids can be expanded in long-term cultures and initiate tumors in immunodeficient mice.

Of note, the organoids may represent an innovative tool for studying tumor-initiating cells [TIC]. TIC is a rare cancer cell population thought to be the engine of tumor formation, relapse, metastasis, and chemoresistance in liver cancer [50]. However, TIC is hard to be cultured in vitro. Liver organoids offer structures resembling an adult organ, favoring TIC survival in in vitro culture, overcoming the major bottleneck in TIC research.

Recently, alternative approaches have been explored, such as combining organoid and CRISPR/Cas9 technology. Two studies reported the generation of genetically modified CC organoids to determine the effects of genetic mutations and provide a platform for mechanistic studies of cancer gene functions in a more in-line, human context [51,52]. This approach would help generate organoids overcoming the limitations exploited by the work of Broutier and Nuciforo [45,48], who demonstrated that liver cancer organoids can be obtained only from poorly differentiated tumors.

Primary liver cancer-derived organoid cultures preserve the histological architecture, gene expression, and genomic landscape of the original tumor, allowing for discrimination between different tumor tissues and subtypes, even after long-term expansion in culture in the same medium conditions [45]. However, the application of organoids has several shortcomings, including high costs, limited availability of origin tissues, and the need for multilineage liver organoids to replicate the true cellular heterogeneity of the liver [53]. Organoids still need several improvements because they are difficult to generate and lack key components of in vivo systems such as blood vessels and the immune system [54].

### 2.3. Organ-on-Chip

In recent years, organ-on-chip (OoC) has become an exciting alternative developed to overcome the drawbacks intrinsic to 2D and 3D in vitro models [16]. These OoC models, combined with improved microfluidics, microfabrication, and tissue engineering manufacturing, represent a powerful tool for patient-specific studies [17]. Indeed, OoCs can be defined as biomimetic platforms replicating human physiology, with features spanning from in vivo-like microenvironments to cell–cell and cell–ECM interactions [18,19]. Moreover, OoCs functionalized with patient-derived cells allow for the setting up of patient-specific platforms to investigate biological mechanisms or can be used in drug screening applications. 

Recently, Lu et al. [55] developed a liver-on-chip better reflecting the tumor microenvironment. For this purpose, they integrated essential components from decellularized liver matrixes with gelatin methacryloyl, obtaining a clear improvement in the performance of this tumor-on-a-chip that was eventually used to investigate dose-dependent drug responses in the presence of acetaminophen and sorafenib. 

In another study, OoCs and microfluidics were used to establish a hypoxic tumor microenvironment model to test cytotoxicity induced by drugs such as paclitaxel and the related mechanism of drug resistance [56]. 

Sharifi et al. [57] reported a liver-chip system whereby the authors elucidated the critical mechanisms responsible for liver cancer invasion. Furthermore, the same platform was used to analyze the inhibitory effect of thymoquinone and its ability to hinder the migration of liver cancer cells into bone tissue [57].

Liu et al. [58] constructed a liver sinusoid platform simulating the Disse space and mimicking the physiological conditions to investigate drug treatments and their response to early and late fibrosis progression.

OoC technology was also utilized to create a multiorgan platform, specifically focusing on the gut–liver axis. The study investigated how the gut microbiota alters irinotecan metabolism by converting its inactive metabolite SN-38G into its toxic metabolite SN-38 [59]. Thus, this model could help to elucidate the interplay between gut microbiota and pharmaceuticals. 

Interestingly, Polidoro et al. [60] created an innovative patient-derived cholangiocarcinoma (CCA)-on-chip platform integrating the major components of the tumor microenvironment (tumor cells, cancer-associated fibroblasts (CAFs), endothelial cells, and immune infiltrate) and, therefore, a more physiological-like CCA niche to make available a precision tool for high throughput screening for anticancer drugs. A similar model would also be highly precious for HCC. 

### 2.4. Precision-Cut Tissue Slices (PCTSs)

Different limitations occurring due to the difficulty of investigating liver pathophysiology using 2D cell lines and organoids could be overcome using precision-cut tissue slices. PCTSs are thin sections of living tissue obtained using specialized cutting tools and techniques. They have emerged as an intriguing approach that provides an ex vivo model recapitulating the features of the tissue of origin. Indeed, PCTSs preserve the cellular heterogeneity and histoarchitecture, including the presence of liver-infiltrating immune cells, mirroring the complexity of the tumor and its microenvironment. PCTSs are reproducible, of low cost, and maintain the viability of the different cells for up to 5 days in standard cultures. Their survival may be extended to 15 days under proper conditions [20,21].

PCTSs derived from liver tumors have been shown to represent a valuable system for the preclinical testing of drug efficacy [22]. For instance, sorafenib was used in the context of liver fibrosis along with several other compounds [61] or regorafenib to assess the reproducibility of combining cryopreservation protocol with HCC tissue slices [62]. Kern et al. [63] applied PCTSs for comparative studies of HCC and the surrounding liver to analyze levels of apoptosis and proliferation after selective COX2 inhibition. Moreover, Zimmermann et al. [64] successfully employed the PCTS HCC model as a predictive test system to evaluate the safety of oncolytic vaccine viruses. Liver slices were also used to recapitulate the phenotypic features of two rare, inherited diseases affecting urea metabolism [65]. Several studies investigated the progression of metabolic-associated steatotic liver disease (MASLD), which eventually ends up in HCC development. In particular, a study by Li et al. [66] aimed to increase the incubation time of PCTS to examine different stages in MASLD to establish a robust and fast ex vivo model and facilitate the identification of novel therapeutic strategies. Finally, PCTSs could also help advance immunotherapeutic approaches for treating HCC. Indeed, several studies have exploited the potential use of PCTSs as a preclinical tool for capturing patients’ heterogeneity and efficaciously developing immunotherapeutic strategies for primary liver cancer [67]. These ex vivo models possess the potential to integrate new treatments based on immune checkpoint inhibitors due to the availability of immune-competent HCC specimens, which allow for the testing of combination therapies and personalized approaches [68].

### 2.5. Scaffold-Based Models

Another tool available to investigate cancer microenvironment interactions that lies somehow halfway between 2D and in vivo models is the scaffold-based system. Unlike other 3D culture methods, scaffold-based models provide a physical matrix in which cells are capable of aggregation, division, and migration [23,24]. Matrigel, collagen, and alginate or synthetic hydrogels (PEG, polyethylene glycol) have been applied to reproduce a culture model in which cell–ECM interactions are present [25]. Leung et al. [69] developed a biocompatible chitosan–alginate 3D scaffold to mimic the in vivo interactions of hepatoblastoma HepG2 cells and tumor microenvironment. In this study, the authors observed differences in proliferation rates when HepG2 cells were compared to 2D cultures. Moreover, the study revealed an increase in levels of angiogenic factors such as interleukin (IL)-8 and vascular endothelial growth factor (VEGF) and pronounced resistance to doxorubicin [69].

Calitz et al. [70] designed an HCC model surrounded by fibrotic stromal compartments and vasculature. Collagen and fibrinogen were incorporated to mimic the biophysical properties of the tumor ECM. Their results confirmed that the 3D model depicts the in vivo situation more accurately than the 2D culture in terms of response to chemotherapy. In another study, Calitz et al. [71] sought to identify changes in tumor behavior based on matrix composition and stiffness. They designed a biomimetic hydrogel scaffold mimicking fibrotic or cirrhotic liver. They demonstrated that altering ECM composition affected tumor behavior, increasing its metastatic potential and response to therapy.

## 3. Animal Models

**Figure 3:**
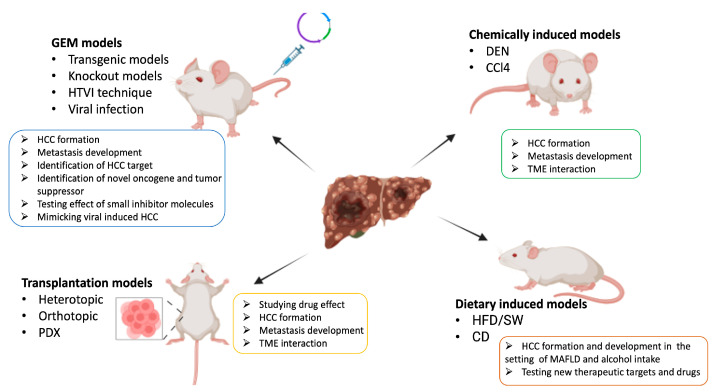
Classification and potential application of HCC mouse models. Abbreviations: CCl4, carbon tetrachloride; CD, choline-deficient; DEN, diethylnitrosamine; GEM, genetically engineered mouse; HCC, hepatocellular carcinoma; HFD, high-fat diet; HTVI, hydrodynamic tail vein injection; MAFLD, metabolic-associated fatty liver disease; PDX, patient-derived xenograft; PLC, primary liver cancer; SW, sugar water; TME, tumor microenvironment. Created with the BioRender tool (www.biorender.com, accessed on 10 June 2024).

The main scope of animal models is to provide an intermediate tool bridging in vitro cell cultures and human clinical trials, allowing researchers to address every aspect of tumor biology within the complexity of a living organism (Figure 3). Considering that mouse genetics are similar to human genetics and gene editing technology has become more accessible to apply, in vivo mouse models are the perfect system to investigate the complex biology of hepatocarcinogenesis—allowing for the evaluation of novel therapies through drug screening—and the crosstalk between hepatic tumor cells and the surrounding environment [72,73]. In this context, a deep knowledge of the phenotypes of different mouse strains is necessary to facilitate decisions during experimental design. Indeed, different strains such as C3H, CBA, and DBA/2 are susceptible to spontaneous or chemical-induced liver tumorigenesis, whereas others, like C57BL/6, BALB/c, and A/J, are resistant to the same oncogenic stimuli [74,75,76].

### 3.1. Chemically and Dietary-Induced HCC Models

The administration of the liver carcinogen diethylnitrosamine (DEN) is a well-established and widely used mouse hepatocarcinogenesis model. In this model, a single intraperitoneal injection of DNA is administered to 14-day-old mice [26]. This event leads to DNA alterations by ethylation and, consequently, the development of neoplastic foci. Eventually, mice develop HCCs within 40 weeks, with a clear male predominance [27,28]. Mouse tumors induced by DEN administration alone frequently harbor initiating activating mutations in either Hras or Braf proto-oncogenes [29]. In particular, Connor et al. [77] identified four recurrently mutated genes that are putative oncogenic drivers of HCC in DEN-treated C3H male mice: Hras, Braf, Egfr, and Apc. Over 80% of DEN-initiated tumor samples carried an activating driver mutation of either Hras or Braf. The remaining ~20% of samples had an activating mutation in the Egfr gene, an upstream inducer of the Ras signaling pathway. These findings strongly suggest that constitutive activation of the Ras/Raf/MEK/ERK molecular cascade is a hallmark feature of this mouse model. Thus, the DEN-initiated mouse model might recapitulate the subset of human HCC characterized by RAS/MAPK signaling dysregulation. In addition, perturbation of the WNT/β-catenin pathway occurs in this model. However, unlike human HCC, commonly harboring activating CTNNB1 mutations, loss-of-function mutations in Apc are predominant in mouse DEN HCC [77].

Carbon tetrachloride (CCl4) has been widely used for decades to induce liver injury and fibrosis in mice [78]. Of note, the susceptibility to CCl4 in mice is strongly strain-dependent, with BALB/c mice being most sensitive to fibrosis induction, whereas FVB/N mice are resistant to the profibrogenic properties of CCl4. Although C57BL/6 mice develop only intermediate liver fibrosis, this strain is frequently used for fibrosis studies in the CCl4 model [78]. The chronic administration of CCl4 and a choline-deficient L-amino-acid-defined diet (CD) for up to 9 months induce NASH with fibrosis and HCC [79]. Tsuchida et al. recently demonstrated that the same diet, combined with low weekly doses of CCl4, triggers a rapid progression to stage 3 fibrosis and HCC within 12 and 24 weeks, respectively. Notably, the resulting liver lesions closely mimicked histological, immunological, and transcriptomic features of human NASH-related HCC, thus representing a rapid induction model suitable to study hepatocarcinogenesis in a clinically relevant setting [80].

Chronic Western high-fat diet and sugar water (HFD/SW) feeding in C57BL/6NJ mice led to significantly increased weight gain, serum and liver lipid levels, liver injury, and glucose intolerance. In addition, mice developed hepatocellular ballooning and liver inflammation; fibrosis occurred at 16 weeks, significantly increased at 32 weeks, and remained elevated at 54 weeks. Notably, liver cancer spontaneously developed in 75% of mice on HFD/SW, half of which were HCC. Furthermore, chronic HFD/SW induced the upregulation of molecular markers of de novo lipogenesis, endoplasmic reticulum stress, inflammation, and the accumulation of p62 [81]. Moreover, the CD+HFD combination promoted the inhibition of weight loss and progression to HCC in about 25% of mice in an NF-κB signaling-dependent manner [82]. However, a significant drawback of this model is the low penetrance of HCC and the lack of information regarding factors that differentiate the few mice that progress to HCC from the majority that remain tumor-free. 

An ideal preclinical model for NASH should be relatively simple, triggered by the same risk factors as in humans (obesity, insulin resistance, and dyslipidemia), and it should match human disease concerning histology, metabolic features, outcomes, gene expression signature, lipid accumulation, and the activation of pathways relevant in humans. The Western diet model, comprised of high cholesterol, high saturated fat, and high fructose, has been shown to promote NASH, characterized by pronounced hepatocyte ballooning and progressive fibrosis after six months of feeding [83]. Asgharpour and colleagues [84] reported a diet-induced animal model of NAFLD-related HCC using an isogenic strain derived from the cross of 129S1/SvImJ and C57BL/6J mouse strains. In this mouse, a high-fat diet accompanied by water consumption ad libitum with a high fructose and glucose content sequentially induces steatosis, steatohepatitis, progressive fibrosis, and HCC. Although most patients with NAFLD have only steatosis without progression, a sizable fraction develops NASH, leading to cirrhosis, HCC, and increased liver-related mortality. In a related model in which tumors were induced using DEN as an initiator followed by phenobarbital as a promoter, chromosomal instability and activating mutations in the β-catenin gene were implicated in tumor progression [85].

In chemically and dietary-induced HCC models, however, the time to tumor formation and the tumor size can vary substantially depending on the mouse strain, and, often, multiple tumor lesions may develop. Thus, these models are difficult to standardize, which results in difficulties in designing and performing experiments, particularly for testing the therapeutic efficacy of new agents. Another concern is the relatively random occurrence of mutations in the DEN-induced HCC model, leading to molecularly heterogeneous tumors.

### 3.2. Heterotopic and Orthotopic Transplantation HCC Models

The most common approach to establishing HCC xenograft models is through either the injection of cancer cell lines or the implantation of tumor tissue. According to the anatomical site, implantation models are divided into heterotopic and orthotopic models. In the heterotopic model, HCC cell lines have often been implanted in mice as subcutaneous xenografts to study HCC growth, invasion, and response to therapies due to the simplicity and reproducibility of the method [30], allowing for tumor volume measurements and monitoring that are easily achieved for anatomic reasons. Orthotopic models are based on the implantation of human cancer cells within their tissue of origin. These models are more advanced in their ability to mimic the tumor microenvironment and precisely reflect the influence of vascularization and stroma with more accuracy; yet, they are more technically challenging [31,72]. However, human HCC cell lines can only be implanted into immunodeficient mice to avoid the rejection of the tumor (transplant). Thus, as these mice lack a functional immune system, it is impossible to investigate the immune influence on the human HCC xenograft model. In addition, the transplanted cells are clonally homogeneous and fully malignant, which hampers the investigation of tumor preneoplastic stage and heterogeneity. Also, in the heterotopic model, the subcutaneous location of the transplanted tumor does not faithfully reproduce the critical HCC–stroma interactions occurring in the liver and the development of local (intrahepatic) or distant metastases [30]. Related to tumor immunology and immunotherapies, syngeneic models based on a recipient mouse with a fully functional immune system are to be preferred. Of interest, a recent work by Qi et al. [32] successfully developed an orthotopic HCC model using an immunocompetent C57BL/6J mouse inoculated with histologically normal oncogenic hepatocytes and treated with CCl_4_ to recapitulate human HCC initiation and progression in the context of an existent liver fibrosis/cirrhosis injury. Similarly, Reiberger et al. [50] generated a syngeneic orthotopic HCC mouse model administering CCl_4_ to induce the development of a cirrhotic liver that mimics the features of human HCC.

### 3.3. Patient-Derived Xenografts (PDXs)

Without prior selection in tissue culture, PDX models can recapitulate human tumor biology more accurately than traditional cell line-derived xenografts. After the National Cancer Institute stopped using the (NCI)-60 panel of tumor cell lines in 2016, the PDX model was established as a more reliable model [33]. PDX can be produced by directly transplanting cancer tissue from patient tumors into immune-deficient mice, such as NOD-SCID and NSG mice, significantly improving xenograft efficiency [34]. Of note, PDX can preserve the original tumor’s genetic, molecular, and histopathologic features and the interaction between the tumor and stroma compartments, thus allowing for the prediction of the patient’s response to therapy. The implanted tumors can be resected and cultured in vitro for further analysis [35]. Nonetheless, some significant limitations should be considered. First, establishing PDX models is costly and time-consuming, which can be prohibitive for personalizing therapies in the setting of an aggressive malignancy. Second, the successful engraftment rate of PDXs is challenging. For instance, PDX mouse models can be established from undifferentiated HCCs, in which the success rate is only approximately 20% [36]. In contrast, samples from patients with O blood type, TNM stage III-IV, and high CEA or CA199 levels are characterized by higher transplantation rates [37]. Moreover, only surgically resected tumors can be implanted and thus do not represent the full spectrum of the disease [86]. Similar to that described in HCC cell xenografts, PDX must be transplanted into the immunodeficient host, thus hindering the investigation of the immune cell effects on the tumor [87]. In addition, the stromal influence on treatment responses may be under-represented in this model [88]. Finally, aggressive tumors have a high chance of successful re-culturing in vitro, which can be rarely achieved in benign or less aggressive tumors [49].

### 3.4. Genetically Engineered Mouse Models

The development of genetically engineered mouse (GEM) models represents a milestone in liver cancer research. Several GEM models have been established, providing a method to study tumor development in an immunocompetent living organism [27]. Several techniques have been developed to create GEM models, including Cre-Loxp recombination, CRISPR-Cas9, and the Sleeping Beauty transposon system. Through these approaches, various models tailored to mimic genetic and epigenetic alterations in human HCC have been generated, allowing for the identification of the precise sequence of events occurring in preneoplastic, early, and late stages of tumor evolution [27,38]. Commonly, GEMs are generated through the activation of oncogenes or the inactivation of tumor suppressor genes to induce tumor formation. Indeed, HCC GEM models generated with single or multiple gene mutations, as well as models that express partial segments of HBV (hepatitis B virus) and HCV (hepatitis C virus) genomes, have been described [39,40].

#### 3.4.1. Transgenic Mouse Models 

The term “transgenic mouse” denotes a mouse in which a normal DNA sequence for a gene is replaced by an engineered sequence or a sequence from another organism. Hepatocyte-specific models were established based on the albumin promoter of Myc [89] and astrocyte-elevated gene-1 (AEG-1) [90]. For example, in GEM models with dual (albumin-driven) AEG-1 and Myc overexpression, mice developed aggressive HCCs and lung metastases. Moreover, DEN treatment substantially accelerated hepatocarcinogenesis in albumin promoter-driven AEG-1/Myc-overexpressing mice [91]. Another mouse model created that co-overexpressed c-myc and TGF-α (Alb-c-myc/MT-TGF-α) was characterized by liver cancer development in 100% of males and 30% of females at 8 months and earlier than the mice that overexpressed c-myc or TGF-α alone [92,93]. The transcription factor E2F1 is commonly upregulated in HCC, and its overexpression in the mouse liver induces dysplasia and tumor development [94]. Moreover, co-overexpression of E2F1 and Myc led to HCC development more rapidly than mice overexpressing the two genes alone, suggesting cooperation during hepatocarcinogenesis [94,95]. GEM models expressing an activated form of β-catenin, the downstream effector of the Wnt pathway, or harboring a liver-specific Apc knockout (KO) showed hepatomegaly or HCC after a long latency [96].

**Figure 4:**
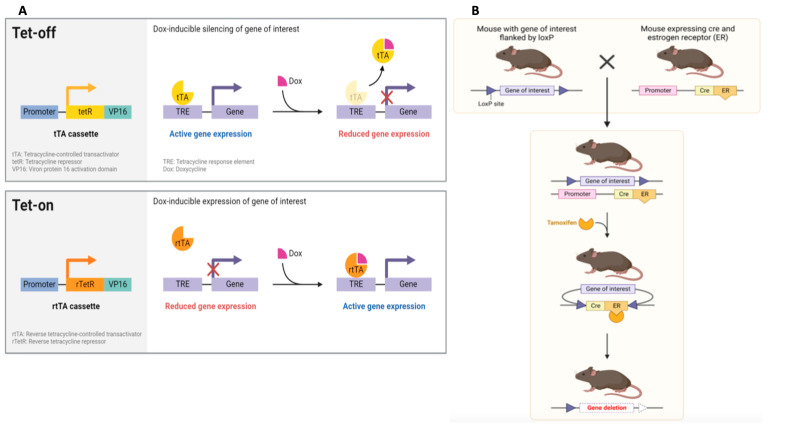
Inducible and conditional GEM models used for spatial and/or temporal control of gene expression. (**A**) Tetracycline (tet)-regulated expression system. Briefly, the tet transactivator acts as a constitutive repressor that is inducibly inhibited by ligands to allow expression from the tet operon (tTA). Alternatively, it functions as an inducible activator of the tet operon upon ligand addition (rtTA). (**B**) Tamoxifen-regulated Cre-loxP system. The Cre-lox tool is primarily used to generate knockout alleles and activate gene expression. The correct insertion of a loxP-flanked “stop” sequence (transcriptional termination element) between the promoter and transgene coding sequence hinders the expression of the gene. In the tamoxifen-inducible Cre-lox model, the system is induced upon tamoxifen administration, and the cells expressing Cre-ER undergo gene inactivation due to the Cre-mediated recombination of the loxP sites, excising the STOP codon in the reporter transgene. Provided by Biorender (www.biorender.com, accessed on 11 June 2024), modified from https://doi.org/10.3389/fphar.2019.00724.

Similarly, liver-specific KO mice are suitable for studying specific gene functions in the liver, including oncogenic and tumor-suppressive effects. The “KO mouse” is a mouse in which the normal gene is missing or engineered in a way that is not transcribed or translated. Traditional, germline KO mice are rarely used because the deletion or inactivation of a given gene is often embryologically lethal. Thus, systems that exert control of target gene expression, such as the tamoxifen-regulated Cre-loxP and the tetracycline (Tet) regulatory systems, are usually preferred (Figure 4). Conditional KO technology is a powerful tool for gene function analysis that allows for region- and time-specific gene manipulation. For example, Katz et al. [97] investigated the role of p53 and whether its deletion is sufficient to determine tumor formation. The authors generated a liver-specific p53 KO model through Cre–Lox recombination. The AlfpCre^+^Trp53^Δ2–10/Δ2–10^ mice developed liver cancer in 14 months, and the tumors were characterized by bilineal differentiation and showed alteration of the retinoblastoma (Rb) checkpoint genes. Shachaf et al. [98] developed a mouse model efficiently using a tetracycline promoter and tetracycline transactivator under the control of a liver-specific LAP promoter to regulate c-Myc expression. These mice expressed c-Myc when not treated with doxycycline (Tet-Off c-Myc model) and developed HCC with a mean latency of 12 weeks. Furthermore, Xiang et al. investigated whether HCC cell expression of myocyte enhancer factor 2D (MEF2D) regulates the expression of PD-L1 in response to interferon-gamma (IFNG) by using mice with liver-specific knockout of MEF2D [99]. In the same way, Li et al. used a liver-specific Setd2 depletion model, finding that Setd2 deficiency is sufficient to trigger spontaneous HCC formation [100]. 

Chronic viral hepatitis and virus-related cirrhosis are the most common risk factors in HCC development [101]. From this perspective, several mouse models have been generated using HBV- or HCV-specific proteins to study hepatocyte injury and malignant transformation [102,103,104]. For example, Chung et al. [105] created a transgenic mouse expressing the HBV polymerase (HBp) or reverse transcriptase (RT) to study the activity related to the pathogenesis of liver disease. All RT mice developed early cirrhosis with steatosis by 18 months, and 10% developed HCC due to a coordinated activation of proapoptotic and proinflammatory responses. Furthermore, Kim et al. [106] used transgenic mice harboring the entire HBx gene to investigate the role of HBV in inducing liver cancer. These mice displayed progressive histopathological changes, starting with multifocal areas of altered hepatocytes, followed by benign adenomas and HCC. Of note, male mice were more susceptible to HCC development and died much earlier than females.

Despite these advantages, however, some critical limitations apply to transgenic mice. Indeed, generating these mice is costly and time-consuming and requires high expertise. Furthermore, the transgene can induce supraphysiologic levels of the gene of interest, leading to unreliable effects (malformations, infertility, etc.). 

#### 3.4.2. Hydrodynamic Transfection Transposon-Based Mouse Models

**Figure 5:**
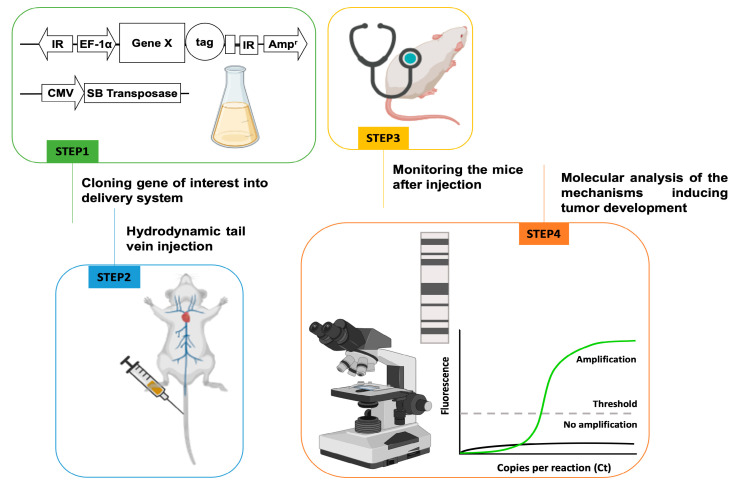
Representative steps in performing the hydrodynamic tail vein injection technique. Modified with Biorender software online (www.biorender.com).

The Sleeping Beauty (SB) transposon system is a synthetic transposon designed to introduce precisely defined DNA sequences into the chromosomes of vertebrates to introduce new traits and discover new genes and their functions. Due to the system’s ability to upregulate or downregulate the levels of a gene or a group of genes, it represents the ideal platform to unravel the effect of overexpression or the inactivation of oncogenes and/or tumor suppressor genes in a given organ [107]. This system has been extensively used in the liver to mimic the pathophysiological and molecular features of human HCC, cholangiocarcinoma, and hepatoblastoma [40,108,109]. Technically, hydrodynamic tail vein injection (HTVI) introduces DNA sequences into the nuclei of up to 40% of hepatocytes upon injecting a large volume of the DNA in saline or lactated Ringer’s solution into the mouse tail vein (Figure 5). Consequently, the solution is delivered rapidly, in less than 10 s, causing a transient, right-sided, venous congestion that engorges and floods the liver with fluid and mechanically disrupts cell membranes. SB has a close-to-random integration profile in the human genome, which contributes to the enhanced safety of SB [108]. This technology has several obvious advantages in stably expressing genes in the liver and establishing novel murine models for liver cancer compared to the traditional transgenic or knockout mouse models. For instance, the injection is performed in 6- to 8-week-old mice, thus not affecting mouse embryonic development. In addition, this technology avoids the generation of costly transgenic or knockout mice and subsequent breeding. Also, it significantly reduces the number of mice needed in the experiments due to high reproducibility. In addition, hydrodynamic transfection can be applied to mice from different genetic backgrounds. Moreover, unlike transgenic mice that express very high levels of the gene(s) of interest, the magnitude of overexpression of the transfected genes is similar to the human disease in livers subjected to HTVI. Furthermore, HTVI allows for the simultaneous injection of several genes, thus permitting the study of gene crosstalk [109], and different plasmids contribute to different oncogenic-driven HCC models. Approximately 25% of HCC tumors present actionable mutations [1] for known oncogenic gene-driven tumorigenesis. Hydrodynamic transfection combination with SB is an appropriate model for performing proof-of-concept studies. The main shortcoming of the transposon methodology is that it specifically targets the mature hepatocyte. Therefore, it is unsuitable for generating models originating from biliary epithelial cells or stem cells [109].

Due to the generation of numerous liver cancer models with this technology, we will only discuss a few of them here. The overexpression of c-MET is found in 20–48% of human HCC samples and represents a potentially therapeutic target [110,111]. In addition, the activated PI3K/Akt/mTOR pathway is closely related to poor differentiation, early recurrence, and poor prognosis of HCC [112]. In this regard, the overexpression of AKT alone via HTVI is sufficient to form liver cancer. Livers from AKT-transfected mice (4 weeks of injection) exhibited a pale and greasy appearance. Microscopically, AKT hepatocytes displayed abundant lipid accumulation in the cytoplasm [113]. After 22–32 weeks of transfection, all AKT mice developed lethal liver cancer. On the other hand, c-met alone failed to engender liver tumors throughout a 1-year observation [114]. Notably, AKT and c-Met co-delivery to the mouse liver rapidly induced liver tumors, triggering the sustained activation of the AKT/mTOR and Ras/MAPK cascades. The lesions displayed the selective upregulation of mTOR targets involved in glycolysis and de novo lipogenesis. Thus, the AKT/c-Met mouse hepatocarcinogenesis model might represent a valid preclinical tool to investigate the therapeutic potential of targeted therapies against HCC subsets characterized by elevated levels of AKT and c-Met [115].

The expression of Pten is reduced in about half of all hepatocellular tumors, leading to the constitutive activation of the PI3K/AKT pathway. Liver-specific knockout of Pten in mice induces lipid accumulation and late-onset liver cancer [116]. Pten deficiency alters baseline glucose metabolism and insulin sensitivity, which is highly linked with the PI3K pathway and thus may mimic some NASH cases. Future studies of PI3K downstream targets to treat patients predisposed to NASH, liver cirrhosis, or HCC could rely on this model. In addition, the sgPten/c-Met murine HCC model shows high levels of mTORC1 and mTORC2 activities and might represent a valid preclinical tool for testing the therapeutic efficacy of novel mTOR inhibitors in HCC [117]. Moreover, when PTEN is deleted, mice exhibit steatohepatitis at 10 weeks of age, fibrosis at 40 weeks, and progression to hepatic adenomas and HCC by 74–78 weeks [118]. However, the mice fail to recapitulate other metabolic syndrome phenotypes, such as obesity and insulin resistance [116,117,118,119]. 

In human HCC, activated mutant forms of PIK3CA occur in about 4% of tumor samples. Somatic mutations of PIK3CA occur around two hotspot regions: the helical domain (exon 9, E545K) and the kinase domain (exon 20, H1047R). Approximately 30–40% of the liver parenchyma of mice transfected with H1047R or E545K was occupied by lipid-rich hepatocytes with an enlarged cytoplasm, displaying hepatic steatosis. This morphology is highly similar to AKT over-expression or Pten deletion in the liver. Of note, the overexpression of H1047R or E545K alone only led to hepatic steatosis with AKT/mTOR signaling activation in the mouse liver, without eventual liver tumor formation. When H1047R or E545K cooperated with NRasV12 or c-Met, liver tumors could form rapidly in these mice. All the tumor nodules in H1047R- and E545K-injected mice displayed the activation of AKT/mTOR and Ras/MAPK cascades [120]. Therefore, this preclinical model is suitable for exploring HCCs with gain of function (GOF) PIK3CA mutations and downstream target screening using small PIK3CA inhibitors such as alpelisib [121].

About 20% of human HCCs may harbor simultaneously Met and β-catenin activation [122]. In human HCC, overexpression and mutation of the MET gene are associated with intrahepatic metastases and vascular invasion, the most clinical determinants of disease outcome [123]. The overexpression of c-Met or the activated mutant form of β-catenin via hydrodynamic injection alone could not promote HCC formation in mice, while their co-expression induced liver tumor development within 6–8 weeks after injection [123]. In particular, Met/β-catenin tumors showed active Wnt and Met signaling with evidence of glutamine synthetase, cyclin D1 positivity, mitogen-activated protein kinase/extracellular signal-regulated kinase, and AKT/Ras/mammalian target of rapamycin activation [124]. This model could be helpful in the preclinical testing of therapeutics directed against the Met and Wnt/β-catenin signaling pathways [114].

c-MYC is overexpressed in up to 70% of viral and alcohol-related human HCCs [125]. Loss of TP53 or MCL1 amplification, leading to apoptosis suppression, often co-occur with c-MYC amplification in human HCCs [126]. To mimic the human HCC cases with the amplification or upregulation of c-MYC, this oncogene was overexpressed in mouse liver. It was found that c-Myc overexpression in the liver is sufficient to induce HCC formation in some susceptible mouse backgrounds [98], while c-Myc overexpression alone induces elevated apoptosis in hepatocytes of resistant mice. Of note, MCL1 is an essential anti-apoptotic gene involved in c-MYC-driven cancer development, and the synergy of MCL1 with c-MYC overexpression could suppress c-MYC-driven apoptosis [127] and trigger liver tumor development [128]. Additionally, over half of HCC cases showed activated mTOR activity [129], which is dominant in c-Myc-dependent hepatocarcinogenesis [128]. Novel MCL1 and mTOR inhibitors could be tested in the c-Myc over-expression HCC model. Also, lipid metabolism is commonly induced in c-Myc-induced hepatocarcinogenesis [130], and lipogenic proteins such as FASN are strongly upregulated. Based on the high frequency of c-MYC overexpression in human HCC, this model may have broad implications for investigating innovative experimental therapeutics targeting lipogenesis in this tumor subset.

Although RAS mutations are rare in HCC, the downstream effectors of the RAS pathway are often activated in HCC. The overexpression of N-RasV12 alone did not induce histological abnormalities in mouse liver. The overexpression of myr-AKT1 induced lipogenesis and hepatocyte proliferation, resulting in liver hepatocellular adenomas ~12 weeks post-injection and, eventually, HCC by 6 months post-injection. Notably, when co-expressing myr-AKT1 and N-RasV12, a tremendous acceleration of liver carcinogenesis occurred. Indeed, all AKT/Ras mice developed a high tumor burden and were required to be euthanized by 5 weeks post-injection. At the molecular level, the mTORC1 downstream effectors in protein translation, angiogenesis, and apoptosis were upregulated in AKT/Ras preneoplastic and neoplastic lesions [131]. Due to the simultaneous activation of the RAS/MAPK and AKT/mTOR pathways in human HCC, this model could be suitable for testing the efficacy of MAPK and mTOR inhibitors on HCC development and progression.

Histopathology of AKT/β-catenin mouse liver tumors revealed the presence of foci composed of vacuolated cells with large cytoplasm consistent with hepatocellular adenoma and hepatosteatosis [132]. Coactivation of the AKT and β-catenin pathways in hepatocytes led to the development of a lipogenic tumor phenotype. Thus, this model portrays the lipogenic phenotype [113] and could help investigate the role of lipid metabolism in HCC formation. 

Loss-of-function AXIN1 mutations and c-Met activation occur in approximately ~3−5% of human HCC. In mice, loss of AXIN1 cooperates with c-Met to induce HCC [133]. While HTVI of sgAxin1 alone did not lead to liver tumor formation, tumors formed rapidly in 9 to 12 weeks after injection when co-transfected with c-Met. In particular, 9 weeks post-injection, the liver parenchyma of sgAxin1/c-Met mice was nearly entirely occupied by large hepatocellular tumors up to 10 mm in diameter, most of them consisting of medium-sized hepatocytes with mild to moderate nuclear atypia. In the human HCC samples, AXIN1-mutant HCCs showed relatively low canonical Wnt pathway activation levels but higher YAP/NOTCH induction [134]. This model could be valuable in investigating candidate treatment efficacy for AXIN1-mutated HCC, such as tankirase inhibitors [135].

## 4. Alternative to Mouse Models: The Chick Embryo Chorioallantoic Membrane

**Figure 6:**
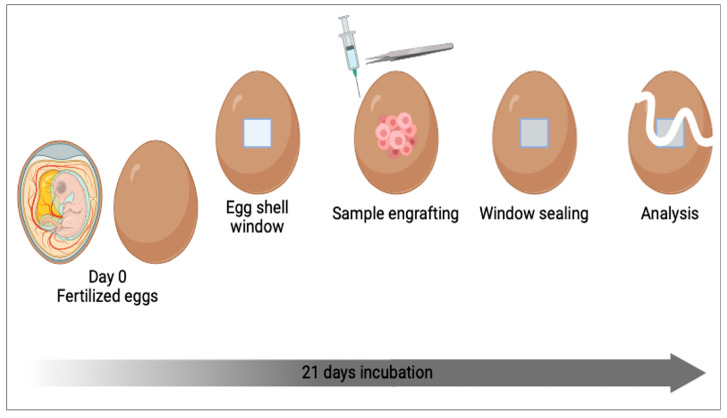
Schematic diagram of CAM assay. CAM, chorioallantoic membrane assay. Modified with Biorender software (www.biorender.com, accessed on 12 June 2024).

The chick embryo chorioallantoic membrane (CAM) is an alternative model to overcome some common limitations encountered with the mouse models already described (Figure 6). Chick embryo development lasts 21 days, and the chorioallantoic membrane forms between days 7 and 12 [136]. The major advantages are that CAM is immunodeficient, highly vascularized, and non-innervated. Therefore, it is an ideal model for xenografting [137]. Regarding its function, CAM can be compared to the mammalian placenta and can be accessed for experimentation by partial removal of the eggshell. Furthermore, CAM expresses several extracellular proteins such as fibronectin, collagen, and laminin, making this model suitable for studying tumor invasion and metastasis. Indeed, the CAM model is a versatile tool to study angiogenesis, drug therapies, tumor development, and other research areas such as wound healing and bone tissue engineering [138]. 

Studying HCC, Li et al. [139] used the CAM model to develop a vascularized tumor that resembled undifferentiated HCC, achieving a 93% survival rate of chick embryos after engraftment. In another study, Eckrich et al. [140] developed a CAM model engrafting the HUH7 HCC cell line to perform routine ultrasonography for repetitive tumor growth and vascularization visualization.

Besides being a versatile and flexible platform for cancer studies, CAM models present some general drawbacks related to the potential non-specific immune response during chick embryo development or the study of metastasis formation due to the short observation period [137]. 

## 5. Utilities of the HCC Preclinical Models

### 5.1. Mechanistic Study

The goal of genomic and transcriptomic profiling is to develop a molecular classification of HCC that identifies driver genes for therapeutic purposes. In particular, a genome-wide screen with CRISPR-mediated genome editing could identify significant genes participating in the formation and progression of liver tumors in vivo. Based on this hypothesis, utilizing the CRISPR–Cas9 system to induce liver-specific genetic knockouts in the murine liver would help elucidate the role of a specific genetic alteration in the development of HCC. In addition, mechanistic studies on the gene crosstalk network could further deepen the understanding of the mystery behind liver tumorigenesis. Moreover, a better understanding of the pathological ability of preneoplastic precursor cells to perceive mediators as tumorigenic growth signals could allow us to discover new opportunities for inhibiting or treating liver cancer.

In addition to the influence of the tumor itself, the immune cells in the tumor microenvironment also play a crucial role in the occurrence and development of tumors. Han Wang et al. used a choline-deficient, high-fat diet, DEN mouse model for NASH-HCC to investigate the immune cell’s influence. They found that the selective increase of intrahepatic Tregs can promote an immunosuppressive environment in NASH livers [141].

### 5.2. Drug Screening

Qiu et al. [142] established the Liver Cancer Model Repository (LIMORE), combining public and newly generated cell lines that represent the genomic and transcriptomic heterogeneity of Eastern Asian hepatocellular carcinomas, and used it to reveal gene–drug associations and potential biomarkers for selecting sorafenib-responding patients. The success rate in establishing human liver cancer cell models was around 50%. Interrogation of the pharmacogenomic landscape of LIMORE discovered unexplored gene–drug associations, including synthetic lethalities, to prevalent alterations in liver cancers. Moreover, this tool could help sorafenib-responding patients select predictive biomarkers. Thus, LIMORE provides a rich resource that facilitates drug discovery in liver cancer.

Laura Broutier et al. obtained surgically resected liver tumor tissue from untreated PLC patients who had no history of viral-meditated hepatitis. They successfully established cultures from tumors derived from eight PLC patients representing the three most common subtypes of PLC: HCC, cholangiocarcinoma (CC), and cholangio-hepatocarcinoma (CHC). They reported C19ORF48, UBE2S, and DTYMK for HCC as novel genes associated with poor prognosis for primary liver cancer. Further studies, though, will be necessary to prove their utility as prognostic factors or their relevance as predictive biomarkers for therapy effectiveness and/or their potential direct involvement in the progression of the disease. These results open up novel opportunities in using tumor-derived organoids for tumor marker discovery [45].

The basic exploration of combined mutated genes with HTVI may simulate more HCC subtypes and optimize drug screening models. For known drug targets, a preclinical model of HCC with corresponding mutations and pathway activation could help to find the most beneficial populations. For unknown drug targets, such as compounds or traditional Chinese medicine, we can investigate the pathway on which the compounds can act depending on multiple mutation models. Thus, for those HCCs with activated related pathways, the drug can be targeted at the subsets. In addition, combination therapy can be investigated in the preclinical models.

### 5.3. Multi-Omics Screening

To stratify multifocal HCC and identify novel diagnostic and prognostic biomarkers by whole genome and transcriptome sequencing, as part of a multi-omics strategy, molecular heterogeneity of hepatobiliary tumor, including intertumoral and intratumoral disparity, always leads to drug resistance. Zhao et al. used scRNA-seq to characterize patient-derived hepatobiliary tumor organoids to explore heterogeneity and evolution. They found inherent variables of transcriptional programs related to cell cycle and epithelial expression across hepatobiliary tumor organoids [143]. Future studies are needed to delineate the heterogeneity of HCC and describe a comprehensive profile further to advance the understanding and precision treatment of HCC.

Combination and verification with multiple HCC models could make the results more convincing. For example, Lei Zhou et al. used both the DEN/CCL4 mouse model and the AKT/N-Ras mouse model to represent chronic fibrotic HCC and rapid steatosis-related HCC to investigate the heterogeneity and properties of Prom1+ cells in HCC [144]. Similar approaches should be employed by comparing several different models of HCC.

## 6. Challenges and Limitations

### 6.1. HBV and HCV Infection and Cirrhosis Features

Cirrhosis is a well-known major risk factor for NAFLD-related HCC [145]. However, the occurrence of NAFLD-related HCC in patients without cirrhosis is increasingly recognized and poses a significant challenge regarding cancer surveillance. Thus, models recapitulating liver carcinogenesis in the presence or absence of liver cirrhosis are highly needed. In this regard, chronic DEN [146] or DEN+CCl4 [147] administration could induce HCC in the background of fibrosis. The addition of CCl4 exacerbated histological features of NASH, fibrosis, and tumor development induced by WD, which resulted in stage 3 fibrosis at 12 weeks and HCC development at 24 weeks [80]. In a syngeneic orthotopic HCC model in immunocompetent mice with liver cirrhosis induced by CCl4, the induction of substantial hepatic fibrosis requires 12 weeks of CCl4 administration. Intrahepatic implantation of mouse HCC cell lines requires 30 min per mouse. Tumor growth varies according to the tumor cell line and mouse strain used. Alternatively, tumors can be induced in a genetically engineered mouse model. In this setting, CCl4 was administered for 12 weeks after the tail vein injection of Cre-expressing adenovirus (adeno-Cre) in Stk4(−/−)Stk3(F/−) (also known as Mst1(−/−)Mst2(F/−); F indicates a floxed allele) mice, and it resulted in the development of HCC tumors concomitantly with liver cirrhosis [50]. In addition, the c-Met/sgPten model also mimicked NASH and exhibited a rapid progression of advanced fibrosis and HCC, with histological, immunological, and transcriptomic features of human NASH. The induction of cirrhosis in mice before the hydrodynamic transfection of oncogenes would be required to study how oncogenes promote tumor development in a cirrhotic microenvironment. The most common approach to inducing inflammation and fibrosis in the liver of mice is by using hepatotoxins by, for example, treating the mice with CCl4 or thioacetamide. In addition, liver fibrosis can also be induced in transgenic mouse models [109].

Less than 20% of HCC cases occur without an underlying chronic liver disease [148]. For HCC without cirrhosis, the DEN-alone-induced model and hydrodynamic transfection combination with the SB model could help test preclinical drugs for HCC without cirrhosis.

Cell line modifications can lead to the susceptibility of tumor cells to HBV. For example, the HepaRG cell line is a valuable tool in drug screening, drug metabolism studies, carcinogenesis, and HBV infection [149]. Human liver organoids could be manipulated to model and study HBV infection, replication, and related tumorigenesis in patient-derived organoids from HBV-infected donors. The liver organoids provide an in vitro mechanistic platform to study the molecular determinants of HBV infection and replication [150]. HBV, by itself, is an inefficient carcinogen. However, it can successfully promote hepatocarcinogenesis initiated by DEN [151]. Liver–SB/HBsAg modifications lead to preneoplastic foci appearing visible from 19.7 weeks of age, followed by hepatocellular adenoma and trabecular HCC. These results are consistent with a model in which the expression of the HBV X protein potentiated the induction of DEN-mediated liver disease [152]. In addition, transgenic mice are an excellent way to study HBV-related HCC. Wu BK et al. generated four lines of HBx transgenic mice, A105, A106, A110, and A112, using the C57BL/6 background [153]. All the HBx transgenic mice spontaneously developed HCC at 13 to 16 months of age. At the histopathological level, the HCC developing in the HBx transgenic mice exhibited a well-differentiated morphology with a trabecular pattern.

Rodents are naturally resistant to HCV infection. One possible model is to leverage immunocompromised SCID mice bearing subcutaneous HCV-infected cell lines. To establish an HCV-related HCC model, an HCV core expression vector was transfected into human hepatoma Huh-7, HepG2, and Hep3B cells to investigate the mechanism of hepatocarcinogenesis by HCV infection [154]. Adel Samson et al. used Huh7-JFH1 cell xenografts carrying a subgenomic HCV replicon (genotype 2a, JFH-1 isolate) to investigate the efficacy of Reo in HCV-associated HCC [155]. The spontaneous HCC model of HCV infection is complicated to achieve solely by HCV infection. Still, other gene losses or modifications can be employed to simulate the HCV spontaneous HCC model. For example, the lack of PML in combination with HCV is associated with increased cell proliferation, fostering tumor development in the liver [156]. Transgenic mice expressing HCV core proteins can develop hepatic steatosis and HCC. HCC developed in approximately 35% of 24-month-old Ppara(+/+):HCVcpTg mice, but no tumors were observed in the other genotypes [157]. PDX is undoubtedly a reliable model for investigating the HCV-related HCC model. Mustafa Nazzal et al. used a PDX mouse model from HCV-associated HCC patients who had undergone resections [158]. The HCV core protein is essential in HCV-related hepatocarcinogenesis because mice carrying the core protein exhibit multicentric HCCs without hepatic inflammation and fibrosis. Three transgenic mouse lineages that contained the HCV core gene were derived (C18, C21, and C49). These transgenic mouse lines had the HCV core gene of genotype 1b under the control of hepatitis B virus regulatory elements. Additionally, 3-, 6-, 9-, and 12-week-old mouse livers displayed marked hepatic steatosis, and 16-month-old livers exhibited gross tumor nodules [104].

### 6.2. Precision Medicine: Generating Models Mimicking the Molecular Subtypes of Human HCC

Human HCC lesions display a high degree of molecular and histological heterogeneity, which is a major reason for the inefficacy of therapies. The HCC phenotype appears to be closely related to particular gene mutations, tumor subgroups, and/or oncogenic pathways [86]. For instance, the activation of the Wnt/β-catenin signaling pathway occurs in 30–50% of cases, caused by mutations in CTNNB1 and AXIN1 genes or APC inactivation. Other frequent mutations or genetic alterations affect TP53, RB1, CCNA2, CCNE1, PTEN, ARID1A, ARID2, RPS6KA3, and NFE2L2 genes, all inducing alterations in cell cycle control. Although driver gene mutations accumulate randomly, specific genes are related to precise molecular HCC subclasses, defined by transcriptomic profiles and histological phenotypes [159,160]. Overall, ~20–25% of patients with HCC have at least one potential actionable mutation per current standards [1]. The HTVI technique would be useful in generating various models, mimicking different molecular subtypes of HCC with unique features. Indeed, the tumors induced through this approach roughly resemble the biological, histological, and molecular characteristics of the corresponding human HCC subsets [161]. Especially, CRISPR-based vectors with sgRNAs are a great way to delete genes affected by inactivating alterations, such as mutations or homozygous deletions, and transposon-based vectors to overexpress genes affected by activating alterations, such as mutations or amplifications.

### 6.3. How to Fit for the Preclinical Study of Immunotherapy

HCCs are perceived as general cold tumors, poorly responsive to current immune checkpoint inhibitors (ICIs). The strategy to convert cold HCC tumors into hot ones may become a promising strategy to improve ICI efficacy. The liver tumor microenvironment is typically characterized by dynamic crosstalk between hepatocytes and immune cells in the natural history of HCC [68]. Concerning further investigation on the efficacy of the novel immune checkpoint inhibitor, humanized mice or GEM models become an appropriate option. HCC-PDX owning the microenvironments could mimic human HCC to a large extent. Humanized mouse models are modified to express human immune cells that invade the tumor, owning more realistic TME [162]. For example, Zhao et al. developed PDX tumors with a type I human leucocyte antigen-matched human immune system in NOD-scid Il2rg−/− (NSG) mice. This model proved a useful parallel-to-human platform for anti-HCC drug testing, especially for immunotherapy [163]. Since the response rate was only ~20% in advanced HCC when treated with immunotherapy, how to select the best subgroup represents a new mission. GEM models could also help choose the subset that responds most to immunotherapy. These models are typically immune-naive, and using artificial antigens in plasmids has even enabled studies of tumor immunology in these SB-HTVI-induced HCC models [161]. 

Antibody–drug conjugates [ADCs] are one of the fastest-growing and most promising oncology therapeutics [35]. More than 60 ADCs are currently in clinical development [35], although ADCs for liver cancer treatment are lacking. Recently, Fu Y et al. established glypican-3 (GPC3) as a potential target for ADC-based HCC therapy. Specifically, they assessed the efficacy of GPC3 conjugated with DNA-damaging agents in HCC based on both HepG2-Luc and Hep3B-Luc xenograft models [164]. Xenograft and peritoneal models help test the effectiveness of novel ADC antibody-related medicines in HCC. 

Another promising biological therapy is adoptive cell transfer, such as chimeric antigen receptor T (Car-T) cells. The efficacy of CAR-T cells in the tumor microenvironment was validated using PDX and murine HTVI HCC models [165]. In addition, Hsiang-Chi Tseng et al. studied the CD147-CAR-modified immune cells for HCC in both xenograft and PDX mouse models [166].

### 6.4. Models to Study HCC Initiation and Progression

Liver tumorigenesis is considered a multistep process, including the activation of proto-oncogenes and disruption of the function of specific tumor suppressor genes [167]. In vitro, the molecular profile of organoids undergoes gradual genetic changes, reflecting the key transforming events from cells that are WT or harbor latent cancer alleles, toward a “premalignant” in vitro-activated phenotype following the introduction of defined genetic alterations until reaching the fully transformed state of malignant tumoroids [52]. Using directly reprogrammed human hepatocytes [hiHeps] and the inactivation of p53 and RB, Sun L et al. established organoids possessing liver architecture and function. HiHep organoids were genetically engineered to model the initial alterations in human liver cancers. Cancer cell lines and organoids derived from primary human cancers recapitulate the mutations and expression profiles of full-blown liver cancers but fail to mimic cancer initiation [168]. Single mouse Lgr5+ liver stem cells can be expanded as epithelial organoids in vitro and differentiated into functional hepatocytes in vitro. A genome-wide screen with CRISPR-mediated genome editing identified significant genes during liver tumor formation [168]. Xenograft-based cell lines and a PDX model recapitulating the mutations and expression profiles of full-blown liver cancers are hard to apply in studying tumor initiation. Still, they are valuable in evaluating the therapeutics’ efficacy in tumor progression. The DEN-induced model can simulate the three processes of liver tumor occurrence, namely, damage, cirrhosis, and tumor. However, tumor initiation in the livers of DEN-treated mice occurs in the context of acute DNA damage; this does not recapitulate the typical clinical presentation of human HCC, which typically arises from chronic inflammatory liver disease causing fibrosis and cirrhosis [169]. HTVI is a valuable tool that resembles the initiation of human HCC [109], which could help understand the gene’s role in initiating hepatocarcinogenesis. 

Less prevalent risk factors for HCC include cirrhosis from primary biliary cholangitis, hemochromatosis, and α1-antitrypsin deficiency. Indeed, patients who develop cirrhosis from hemochromatosis are at an exceptionally high risk of HCC, up to 45% [170]. For these specific subgroups, the ideal model is still lacking.

### 6.5. In Silico Models, Artificial Intelligence, and Machine Learning

Over the years, computational modeling has become a potential alternative to traditional preclinical models and how to perform laboratory-based research. Indeed, in silico experimentation, defined as coupling computer-based technology and software with biology, aims to meet the 3R (Replace, Reduce, and Refine) principles to limit the use of animals and the costs and effort associated with conducting preclinical studies [171,172]. Artificial intelligence (AI) is a technology that enables computers to simulate human intelligence and problem-solving capabilities, in other words, to demonstrate critical thinking and intelligent behavior [173,174]. AI is often mentioned together with machine learning (ML) and deep learning (DL). These subcategories encompass the development of AI algorithms to manage a considerable amount of example data, modeled after the decision-making processes of the human brain, to learn how to make associations and then apply statistical models to data with these learned associations, leading to more accurate predictions over time [175].

One of the areas where in silico models and computational methods best apply is represented by drug design and development, with the great promise to reduce the production pipeline and associated costs. Moreover, in silico models are essential in facilitating the repurposing of FDA-approved compounds, especially in oncology drug discovery [176]. Another approach is to perform, with the help of ML and DL, a combined evaluation of different acquired data, such as clinical, histological, and radiological data, to predict several outcomes in terms of early cancer diagnosis, pathological features, possible response to therapy, and survival rate [177].

Liu et al. [178] used computational models through the Connectivity map (Cmap) database to predict that sorafenib had the potential to inhibit the activity of histone deacetylase (HDAC). Afterward, in vitro experiments confirmed that sorafenib indirectly inhibited HDAC activity in both sorafenib-sensitive and -resistant cells, proving the efficacy of in silico analysis in studying polypharmacology. In another study, Sun et al. [179] used in silico modeling to identify potential biomarkers and novel targets for HCC treatment. They identified DEP domain-containing protein 1B (DEPDC1B), not reported to be associated with HCC, that could be useful as a new biomarker for diagnosis and prognosis.

In summary, the use of AI and machine learning models such as gradient boosting, deep learning, and random forest have outperformed traditional statistical methods, contributing to ameliorating detection, diagnosis, prognosis, and risk prediction capabilities in cancer research, thus making possible early detection and precision management. However, a crucial step is the integration between in silico and in vivo approaches in order to induce a change in cancer research and move towards a more human-centric model of the disease to deliver accurate, personalized prognostic assessments to support clinical decision-making. At the moment, from the evidence provided by the scientific literature, AI could be profitably used to complement and guide in vivo experiments and, under the principle of the 3Rs, lead to the more efficient and effective use of animals in preclinical research.

## 7. Discussion

In summary, constructing the ideal model of HCC is based on five aspects to consider: (1) Etiology. Simulate the original etiology and track the occurrence and development of HCC, which is conducive to the early intervention and diagnosis of liver cancer. (2) Recoverability. Restore the natural microenvironment of liver cancer and the interaction network between immune cells and tumor cells, which helps target those immunosuppressive cells that support the growth of tumor cells, causing death. Studying hepatocarcinogenesis requires GEM models with the induction of specific mutations for a reproducible expression of HCC to mimic human disease. PDX can simulate the complex natural environment of patient tumors. PDX models in maelstrom contributions to treatment responses may be under-represented. GEMs can, to some extent, reflect tumors caused by a series of oncogenic driving genes. The DEN-induced model leads to tumor mutations that do not match the pathological type of human HCC mutations. Subcutaneous xenograft models fail to take the liver microenvironment into account. (3) Operability. The subcutaneous injection of an HCC cell line might help study response to therapy, as tumors are easily accessible, and growth can be monitored with serial tumor measurements. An orthotopic injection of HCC cells may require a surgical procedure, which might be technically challenging [72]. (4) Success rate, time to build the model, price, and techniques. For example, the DEN-induced HCC model achieves 30% incidence at 9 months, while DEN and CCl4 trigger liver tumor development in 100% of mice by 5 months of age [180]. Xenograft and GEM models need several weeks to be established. Orthotopic injection of HCC cells might be used, but this often requires a surgical procedure, which may be technically challenging. Transgenic mice are both cost-consumi ng and time-consuming. (5) Reflecting the heterogeneity of liver cancer. The heterogeneity of liver cancer has always been a considerable obstacle in the treatment of liver cancer. DEN-induced HCC had a certain degree of heterogeneity, but with genetic differences compared to human HCC. Needle HCC PDX reflected the comprehensive landscape of liver cancer tumors to some extent. The HTVI methodology could generate models that mimic different molecular subtypes of HCC with common driver mutations. It is not ideal to reflect the spatial and temporal heterogeneity within the tumor in the current models. The available models can show the therapeutic efficacy to a certain extent to satisfy the need of preclinical research and provide meaningful evidence to guide clinical trials.

Drug toxicity is a concern for subsequent FDA-approved therapy based on clinical trials. In 2023, the FDA canceled the requirement of animal models for new drugs. In the past, for a drug to be approved in the United States, the FDA typically required toxicity tests on one rodent species, such as a mouse or rat. However, most drugs fail in clinical trials because they are unsafe or ineffective, so the necessity for animal experiments is questioned [181]. However, the results of preclinical model experiments will undoubtedly enlighten clinical trials. A deep understanding of the molecular pathogenesis and heterogeneity of HCC could optimize clinical trial design. Potential effective drug combination strategies should be evaluated in a model with specific mutations to screen the population with the most benefit for future clinical trials, thus optimizing the inclusion criteria for clinical trials and integrating resources. Importantly, as mentioned above, HCC mouse models play significant roles in mechanistic studies and drug and multi-omics screening.

Overall, no single mouse model can capture all aspects of human HCC, but each can recapitulate some disease features. Suitable models can be selected based on experimental research purposes and scientific assumptions. These models can help delineate resistance mechanisms to current therapies and lead to personalized medicine tailored to individual patient needs. Integrative multi-omics (proteomics, genomics, epigenomics, transcriptomics, metabolomics, and peptidomics) approaches could be applied to discover novel biomarkers to improve the sensitivity and specificity of HCC early and accurate diagnosis. Furthermore, new drugs can be screened on these models to bring hope to patients with HCC. Also, these mouse models will help to identify the most beneficial subset featured with genomic characteristics and predict molecular biomarkers related to drug sensitivity or resistance.

## 8. Conclusions

The contribution given by experimental in vitro and in vivo models to HCC research is remarkable and undeniable. Indeed, these models have played a critical role in unraveling HCC molecular pathogenesis and potential therapies. Therefore, these classical approaches will continue to support scientific discovery in different medical fields, not limited to liver cancer. Nonetheless, the various models exhibit important limitations (lack of tumor heterogeneity, absence of a proper tumor microenvironment and/or the immune milieu, paucity of metastatic models, etc.) that should be overcome. As an ideal model does not exist, it is up to us, the researchers, to select the most appropriate system, either alone or in association with other tools, to reach the planned goal(s). This selection process should follow established scientific criteria and standards and strictly adhere to the 3R (Replace, Reduce, and Refine) principles. In these terms, the application of the newly developed in vitro techniques such as OoC and microfluidics, PCTS, and bio-scaffold, together with the integration of AI and machine learning, could increase the potency of classical models, accelerate scientific progress, and minimize the use of animals.

## Figures and Tables

**Table 1:** Overview of preclinical models used for hepatocellular carcinoma research.

In Vitro Model.	Comments.	Advantages	Disadvantages	Refs.
2D cell lines	Valuable preclinical model routinely used in cancer research and drug discovery.	Easy and low maintenance costs; highly reproducible; variety of cell lines commercially available.	Cultures are not in 3D; poor retention of histological and mutational landscape from the original tumor; single cell type with limited cell-cell interactions.	**[11,12,13]**
Organoids	Provide better insights into cellular activities due to advanced 3D architecture.	Forms a 3D structure; can be patient-derived; predicts patient clinical outcomes; facilitates mechanism of action studies; cost-efficient; Suitable for high-throughput screening.	Long time to develop; lack of specific cell types, including immune cells; technically challenging.	**[14,15]**
Organs on Chips	A combination of cell biology, engineering, and biomaterials technology.	Biochemical and physical resemblance of in vivo TME; high levels of control of the TME; multiple cell types can be co-cultured.	Does not mimic ECM found in vivo; high-cost materials; lack of standardization in design, manufacturing, and use.	**[16,17,18,19]**
Tissue Slice Cultures	Thin sections of living tissue obtained using specialized cutting tools and techniques.	Maintains the complex structure of tissues found in vivo; multiple readouts analyzed; suitable replacement for animal models.	Long and difficult preparation procedure for culturing; limited culture time to investigate chronic diseases.	**[20,21,22]**
Bio-Scaffold	Prefabricated 3D structures composed of natural compounds or synthetic materials.	Physical support for cell proliferation, and migration; designed to resemble the in vivo ECM; drug screening.	Composition heterogeneity of the materials; verification of materials’ properties; reduced reproducibility.	**[23,24,25]**
**In Vivo Model**				
Chemically and Dietary induced	Chemical carcinogens administered byintraperitoneal injection or introduced with a modified diet.	Can be administered orally; provides a reproducible model of HCC progression; higher heterogeneity; easy to associate with other methods that promote HCC; dietary model better tool for modeling NASH/NAFLD.	Species difference from humans; time to develop HCC depends on sex, age, and strain; not suitable for high-throughput screening.	**[26,27,28,29]**
Xenograft	Utilizes human HCC cell lines; injection into the subcutaneous tissue (heterotopic) or directly into the liver (orthotopic).	Monitor tumor growth in real time; shows cell-cell interaction; heterotopic engraftment is relatively cost-effective and easy procedure; orthotopic occurs in the natural liver microenvironment and mimics closely HCC development.	Heterotopic implantation is not an accurate physiological representation lacking the microenvironment; requires an immune deficient host; orthotopic engraftment is difficult to perform; high maintenance; not suitable for high-throughput screening.	**[30,31,32]**
Patient Derived Xenograft	Utilizes human HCC tumor tissue.	Can be used to test personalized therapies; retains characteristics of donor tumor; recapitulates the original tumor reflecting morphology, genetic mutations and correlates with patient outcome.	Species difference from humans; immunodeficient mice; high maintenance cost; not suitable for high throughput screening; limited by patient samples.	**[33,34,35,36,37]**
Genetically Modified	Single or multiple gene manipulation; activation of oncogenes or inactivation of tumor suppressor gene; tumors develop from normal cells and are homogeneous in the genomic aspect.	Enables study of hepatocarcinogenesis; includes natural TME; allows testing of targeting gene therapies.	Costly; species difference from humans; manipulated genes might be vital for embryo development; single gene HCC models usually have a longer latency for tumor formation.	**[27,38,39,40]**

Abbreviations: HCC, Hepatocellular carcinoma; TME, Tumor microenvironment; ECM, Extracellular matrix; NASH, Non-alcoholic steatohepatitis; NAFLD, Non-alcoholic fatty liver disease.

**Table 2:** HCC cell lines publicly available and described in the literature as established in vitro experimental models.

Cell Line	Origin	Morphology	Etiology and Other Characteristics	Applications
HepG2	European male 15 years old	epithelial	Originally thought to be an HCC cell line but shown to be from an hepatoblastoma	3D cell culture, Cancer research High-throughput screening, Toxicology
Hep3B	African male 8 years old	epithelial	HCC with integrated HBV genome fragment (2,3 Kb size)	3D cell culture, Cancer research, High-throughput screening, Infectious disease research, STD research, Toxicology
HUH7	Asian male 57 years old	epithelial	Derived from a well differentiated HCC; Presence of several plasma proteins; Do not express TP53	3D cell culture, Cancer research, in vitro drug metabolism and toxicology evaluations
SMMC-7721	Asian female 30 years old	epithelial-like	Contaminated. Originally thought to originate from HCC but shown to be a HeLa derivative	3D cell culture, Bioproduction
SNU182	Asian male 24 years old	epithelial	Pleomorphic; Tumor grade: III-IV; HBV DNA detected by Southern blot hybridization; HBV genomic RNA was not expressed	3D cell culture, Cancer research, Infectious disease research, STD research
SNU387	Asian female 41 years old	epithelial	Pleomorphic; Tumor grade: IV-V; HBV DNA detected by Southern blot hybridization	3D cell culture, Cancer research, Infectious disease research, STD research
SNU449	Asian male 52 years old	epithelial; diffusely spreading cells	HCC; Grade II-III/IV; HBV DNA detected by Southern blot hybridization; HBV genomic RNA was not expressed	3D cell culture, Cancer research
SNU475	Asian male 43 years old	epithelial	Derived from primary HCC; Tumor grade: II-IV/V; HBV DNA detected by Southern blot hybridization; HBV genomic RNA was not expressed	3D cell culture, Cancer research, Infectious disease research, STD research
MHCC97H	Asian male 39 years old	epithelial	Primary HCC; High metastatic potential	3D cell culture, Cancer research
PLC/PRF75	African male 24 years old	epithelial	HCC derived with presence of hepatitis virus B surface antigen (HBsAg); Reduced expression of TP53	3D cell culture, Cancer research, Infectious disease research, STD research
HLF	Asian male 68 years old	epithelial	Derived from a well differentiated HCC	3D cell culture, Cancer research, in vitro drug metabolism and toxicology evaluations

Abbreviations: HCC, Hepatocellular carcinoma; HBV, Hepatitis B virus; STD, sexually transmitted disease. All the data listed have been cross-referenced among “Cellosaurus” (https://www.cellosaurus.org, accessed on 10 June 2024), “The human protein atlas” (https://www.proteinatlas.org/humanproteome/cell+line/liver+cancer, accessed on 10 June 2024), the American Type Culture Collection “ATCC” (https://www.atcc.org, accessed on 10 June 2024) and Japanese Cancer Research Resources Bank “JCRB cell bank” (https://cellbank.nibiohn.go.jp/english, accessed on 10 June 2024).

## Data Availability

Data sharing is not applicable. No new data were created or analyzed in this study.

## Abbreviations

2D, two-dimensional; 3D, three-dimensional; ADC, antibody–drug conjugates; AEG-1, astrocyte-elevated gene-1; AI, artificial intelligence; APC, adenomatous polyposis coli protein; ARID1A, AT-rich interaction domain 1A; CA199, carbohydrate antigen 19-9; CAF, cancer-associated fibroblasts; CAM, chorioallantoic membrane; CCA, cholangiocarcinoma; CCl4, carbon tetrachloride; CD, choline-deficient; CEA, carcinoembryonic antigen; COX2, cyclooxygenase-2; CRISPR/Cas9, clustered regularly interspaced short palindromic repeats/CRISPR-associated protein 9; CTNNB1, catenin beta 1; DEN, diethylnitrosamine; DEPDC1B, DEP domain-containing protein 1B; E2F1, E2F transcription factor 1; ECM, extracellular matrix; EGFR, epidermal growth factor receptor; FASN, fatty acid synthase; GEM, genetically engineered mouse; GOF, gain of function; HBsAg, hepatitis B virus surface antigen; HBV, hepatitis B virus; HCC, hepatocellular carcinoma; HCV, hepatitis C virus; HDAC, histone deacetylase; HFD, high-fat diet; HTVI, hydrodynamic tail vein injection; IFNG, interferon-gamma; IL, interleukin; iPSC, induced pluripotent stem cell; KO, knockout; LGR5, leucine-rich repeat-containing G-protein coupled receptor 5; LIMORE, Liver Cancer Model Repository; MAFLD, metabolic-associated fatty liver disease; MAPK, mitogen-activated protein kinase; MASLD, metabolic-associated steatotic liver disease; MCL1, myeloid cell leukemia 1; MEF2D, myocyte enhancer factor 2D; NAFLD, non-alcoholic fatty liver disease; NASH, non-alcoholic steatohepatitis; NOD/SCID, non-obese diabetic/severe combined immunodeficiency; NSG mice, NOD/SCID gamma mouse; OoC, organ-on-chip; PCTS, precision-cut tissue slice; PD-L1, programmed death-ligand 1; PDX, patient-derived xenograft; PEG, polyethylene glycol; PI3K, phosphoinositide 3-kinase; PLC, primary liver cancer; Rb, retinoblastoma; RT, reverse transcriptase; SB, Sleeping Beauty; STD, sexually transmitted disease; SW, sugar water; TERT, telomerase reverse transcriptase; TGF-a, transforming growth factor alpha; TIC, tumor-initiating cells; TME, tumor microenvironment; TP53, tumor protein 53; VEGFR, vascular endothelial growth factor receptor.
